# Severe exacerbation of facial dermatitis with swelling following introduction of abrocitinib in a patient with atopic dermatitis

**DOI:** 10.1186/s13223-024-00911-w

**Published:** 2024-09-19

**Authors:** Shirui Chen, Chongtu Yang, Yonghong Lu

**Affiliations:** 1 Department of Dermatovenereology, Chengdu Second People’s Hospital, Chengdu, Sichuan China; 2 Department of Radiology, West China Hospital, Sichuan University, Chengdu, Sichuan China

**Keywords:** Abrocitinib, Facial dermatitis, Atopic dermatitis, Provocation test, JAK1 inhibitor, Adverse events

## Abstract

**Background:**

Abrocitinib, an oral small-molecule Janus kinase 1 (JAK1) inhibitor, has been widely accepted for the treatment of moderate-to-severe atopic dermatitis (AD). Currently there is a paucity of data on the adverse events (AEs) after abrocitinib treatment, especially on rare events such as exacerbation of facial dermatitis, and their causal relationship and subsequent management remains poorly elucidated.

**Case presentation:**

A 43-year-old female patient with moderate AD received dupilumab after failure of topical treatments. Facial dermatitis persisted and became refractory after dupilumab treatment, and the patient changed treatment to oral abrocitinib. Fifteen hours after the first dose of abrocitinib, she developed exacerbation of facial dermatitis with swelling. The patient was initially diagnosed as abrocitinib-induced hypersensitivity. However, a score of 3 of the Naranjo adverse drug reaction assessment indicates week correlation between abrocitinib therapy and exacerbation of facial dermatitis, and negative results from subsequent drug provocation test further suggests no causal relationship.

**Conclusions:**

The present case report highlights the necessity of careful determination of abrocitinib-induced hypersensitivity, which should not be diagnosed simply based on the time sequence between drug exposure and symptom occurrence. In addition, caution should be exercised for drug withdrawal, especially when confirmative evidence is absent. Drug provocation test can be helpful and effective treatments could be continued unless severe AEs occur.

## Background

Atopic dermatitis (AD) is a common chronic inflammatory skin disease associated with persistent itch, with a reported prevalence of over 10% among adults and 15–20% among children, and remains a global health challenge [1, 2]. Treatment for AD is mainly determined based on its clinical stage (mild, moderate, or severe), and systemic therapy is widely recommended for moderate-to-severe AD refractory to topical treatments [3]. Abrocitinib, a small-molecule Janus kinase 1 (JAK1) inhibitor, demonstrated superior efficacy in reducing itch and other disease signs of AD than dupilumab in patients with moderate-to-severe AD [4, 5]. More importantly, a recent phase 3 clinical trial supports the efficacy and safety of abrocitinib in patients with prior dupilumab treatment, regardless of the response status of using dupilumab [6].

Since abrocitinib is a rather novel therapy compared to dupilumab, data on treatment-related adverse events (AEs), especially rare AEs, are limited in the real-world situation, resulting in discrepancies on subsequent management. Exacerbation of facial dermatitis after abrocitinib has sparsely been reported, and the underlying mechanisms remain poorly elucidated. Consequently, differential diagnosis with allergic- and phototoxic-contact facial dermatitis and subsequent management remain an unmet need.

## Case presentation

**Fig. 1:**
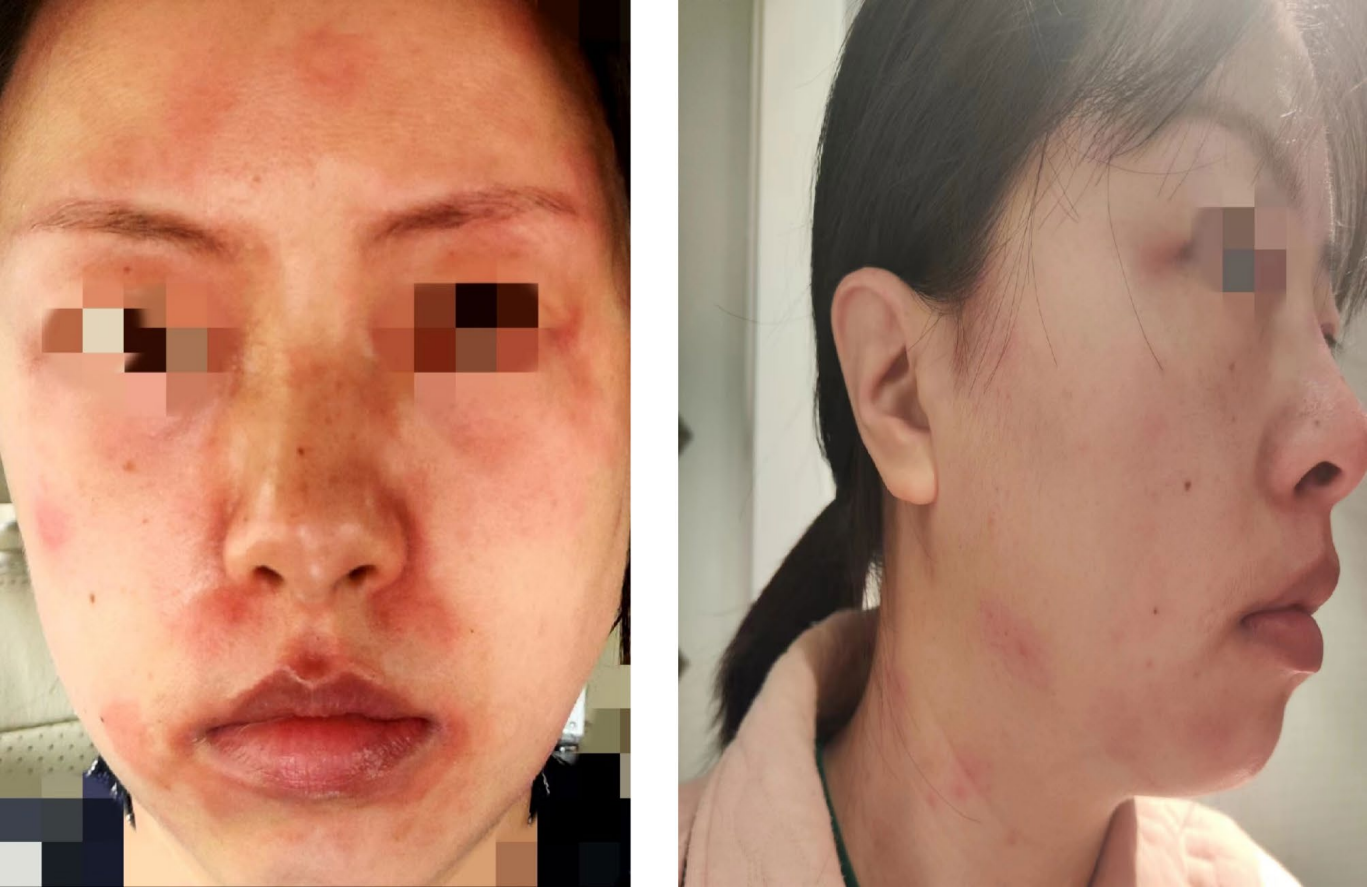
Scattered erythema occurred on the face and neck after the third dose of dupilumab injection in the patient with moderate atopic dermatitis

A 43-year-old female with moderate AD presented to our clinic because of recurrent general erythematosquamous papules and plaques for more than one year. The patient was previously diagnosed as AD according to local guidelines [7]. She received long-term conventional treatments regularly, including topical glucocorticoid and antibiotics, and oral antihistamine, but achieved limited effects. Afterwards, she started to receive subcutaneous injection of dupilumab with a total of 10 doses for 4 months. General rash and itch achieved remission, but new facial dermatitis developed and became refractory after the third dose of dupilumab (Fig. 1). Thus, treatment regimen was switched to abrocitinib after finishing the 4-month dupilumab treatment and then a drug withdrawal interval of about 50 days. The patient developed lip swelling 6 h after receiving the first dose of abrocitinib (with a dose of 100 mg), and after 15 h she developed facial swelling, erythema and exudation with burning sensation.

**Fig. 2:**
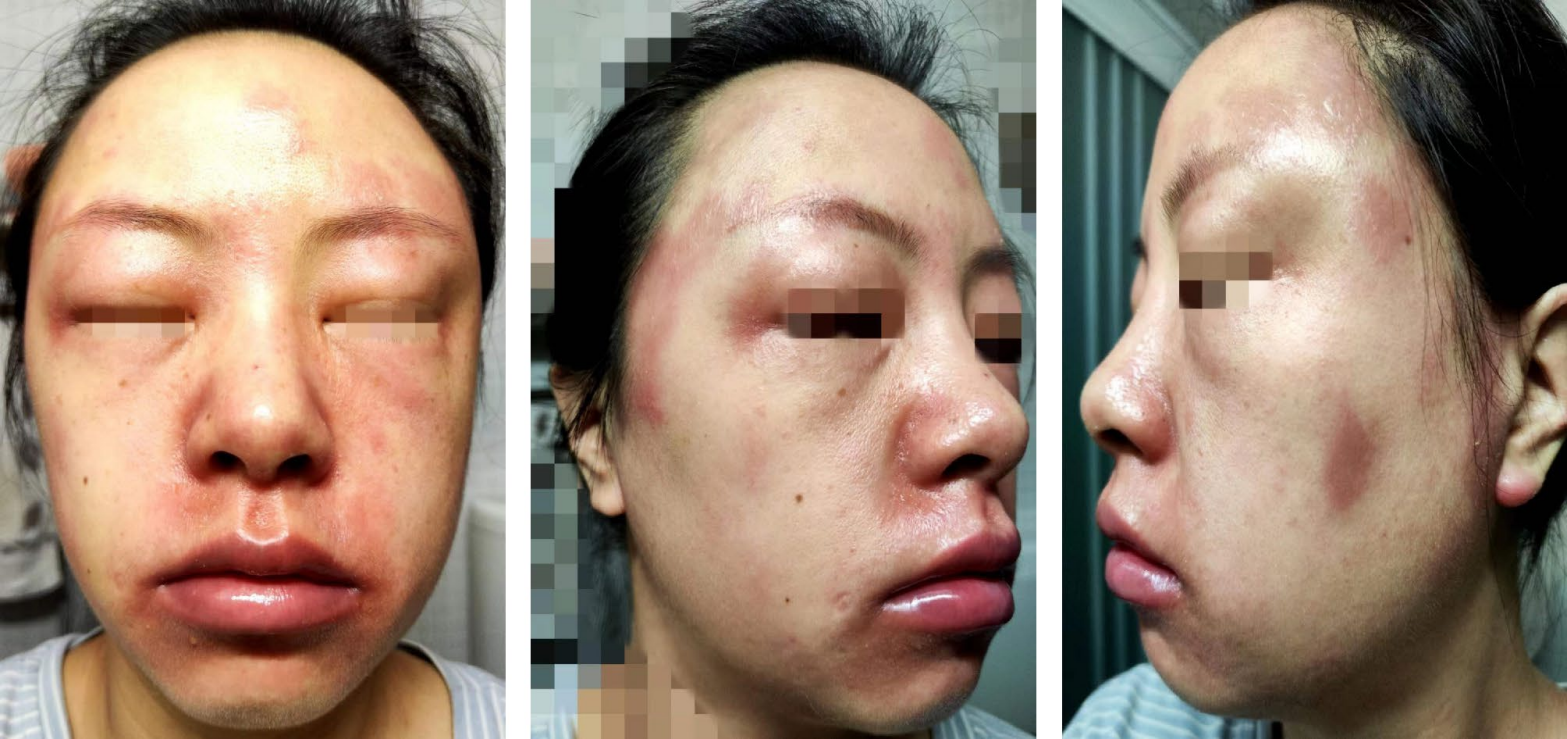
Exacerbation of facial dermatitis with swelling occurred 15 h after initiation of abrocitinib treatment, manifested as diffuse facial swelling, erythema and exudation on the forehead and nostrils

The patient denied any known allergens, did not use new skincare products or other drugs, and was not exposed to significant sunlight. Physical examination found diffuse facial swelling, erythema and exudation around the eyes, nostrils and forehead (Fig. 2). Laboratory tests showed a change of serum eosinophil count from 0.57 × 10^9^/L before abrocitinib treatment to 0.65 × 10^9^/L after treatment. Antinuclear antibody levels were both 1:320 (speckled pattern) before and after treatment, and anticentromere antibody levels were both strongly positive (+++). UVA and UVB-MED tests indicated mild photosensitivity. Other laboratory results including assessment of coagulation, liver, kidney and thyroid function, Interleukin-6 level and rheumatoid factors were within the normal range. The patient was initially diagnosed as abrocitinib-induced exacerbation of facial dermatitis, and according to the World Allergy Organization (2024) recommendation [8], reaction of the patient could be classified as delayed effects (i.e., after the first 30 min of drug administration). The patient was then treated with intravenous dexamethasone and the symptoms improved after one week of treatment. After one month she resumed using dupilumab.

**Fig. 3:**
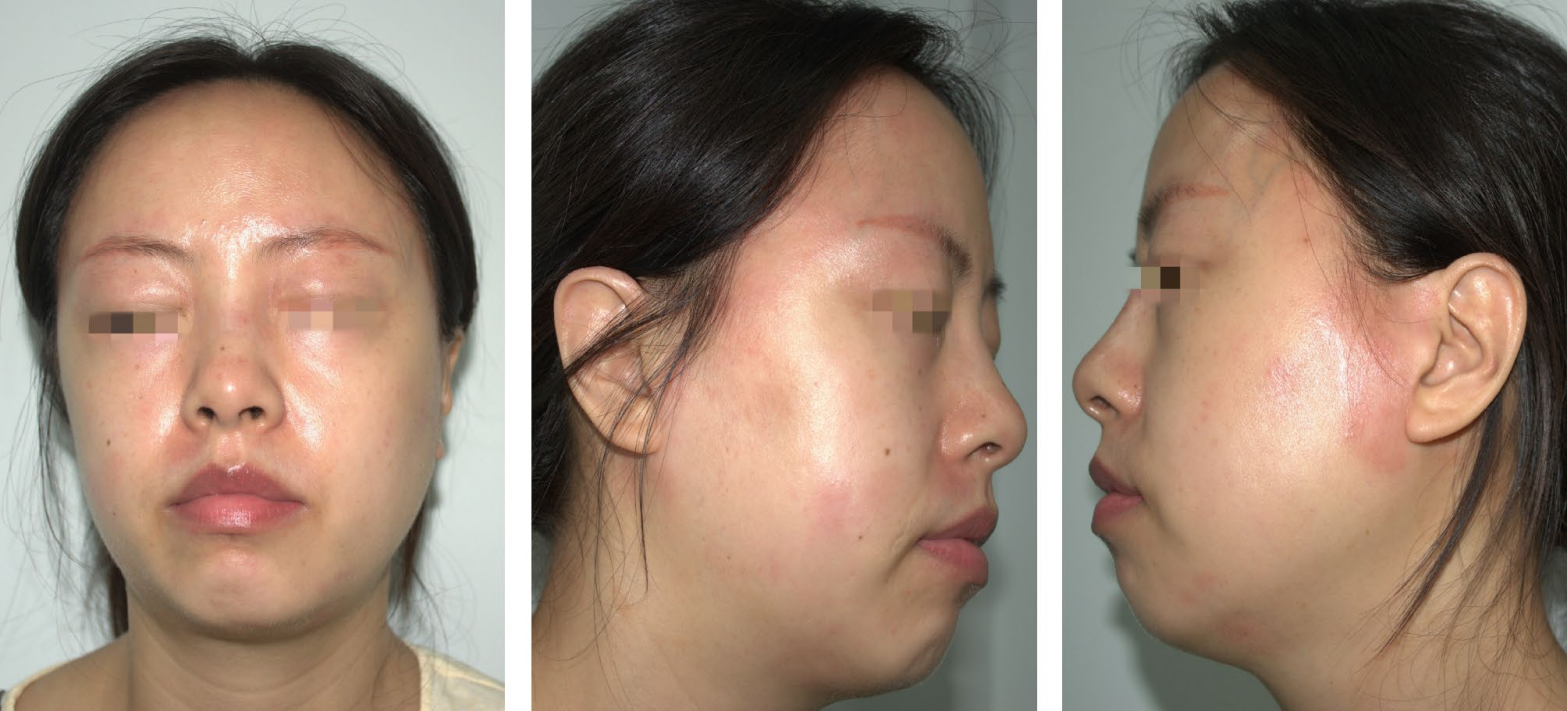
Persisted non-pruritic facial erythema with burning sensation when returned to dupilumab treatment

However, facial erythema with burning sensation occurred persistently and posed substantial burden (Fig. 3). Since a score of 3 was determined using the Naranjo adverse drug reaction assessment [9], there was no conclusive evidence on abrocitinib-induced hypersensitivity. Thus, the patient received drug provocation test for further diagnosis. Abrocitinib tablet was administered orally at incremental doses (10 mg, 50 mg, 100 mg) every 3 days. No exacerbation of facial dermatitis or new rashes were observed during the test, indicating that the aforementioned exacerbation of facial dermatitis was not caused by abrocitinib.

## Discussion and conclusions

Abrocitinib is a highly selective JAK1 inhibitor that effectively blocks the activity of multiple enzymes and inhibits downstream inflammatory pathways, which has been recommended for the treatment of moderate-to-severe AD [10]. Despite its better efficacy compared to dupilumab, abrocitinib is associated with higher incidence of treatment-related AEs [4, 5]. The most common AEs following abrocitinib included nausea, headache and acne [4]. Until now there is only one patient reported to experience angioedema after abrocitinib in a clinical trial [6], which is similar to our case but without any detailed information provided. In real-world situation, there is no patient reported to experience exacerbation of facial dermatitis until now. In our center, a 75-year-old male and a 51-year-old female were also observed to develop facial dermatitis within few hours after receiving abrocitinib. They refused to receive systemic corticoid therapy and continued to use abrocitinib, whose inflammation was gradually controlled and a dose of 100–200 mg per week could effectively control rash and itch. Accordingly, we hypothesized that the poor response and rapid progression of the reported case may partially be attributed to the long course of AD, severe inflammation and insufficient dosage of the initial abrocitinib.

This case report illustrates that abrocitinib could be safely resumed or continued in patients experiencing facial dermatitis shortly after its introduction as this phenomenon is unlikely to represent a hypersensitivity reaction.

## Data Availability

No datasets were generated or analysed during the current study.

## Abbreviations

AD: Atopic dermatitis
JAK1: Janus kinase 1
AD: Adverse event
